# Hydrogen-Deuterium Exchange Mass Spectrometry Reveals a Novel Binding Region of a Neutralizing Fully Human Monoclonal Antibody to Anthrax Protective Antigen

**DOI:** 10.3390/toxins14020092

**Published:** 2022-01-25

**Authors:** Mulin Fang, Zhe Wang, Kathleen Norris, Judith A. James, Si Wu, Kenneth Smith

**Affiliations:** 1 Department of Chemistry and Biochemistry, University of Oklahoma, 101 Stephenson Parkway, Norman, OK 73019, USA; mulin.fang-1@ou.edu (M.F.); xmuwangzhe@gmail.com (Z.W.); 2 Department of Arthritis and Clinical Immunology, Oklahoma Medical Research Foundation, 825 NE 13th St., Oklahoma City, OK 73104, USA; kathleen-norris@omrf.org (K.N.); Judith-James@omrf.org (J.A.J.); 3 Department of Microbiology and Immunology, Oklahoma University Health Sciences Center, 940 Stanton L. Young Blvd, Oklahoma City, OK 73104, USA; 4 Department of Medicine and Pathology, Oklahoma University Health Sciences Center, 1000 Stanton L. Young Blvd, Oklahoma City, OK 73104, USA

**Keywords:** anthrax, antibody, HDX, mass spectrometry

## Abstract

Anthrax vaccine adsorbed (AVA) containing protective antigen (PA) is the only FDA-approved anthrax vaccine in the United States. Characterization of the binding of AVA-induced anti-PA human antibodies against the PA antigen after vaccination is crucial to understanding mechanisms of the AVA-elicited humoral immune response. Hydrogen deuterium exchange mass spectrometry (HDX-MS) is often coupled with a short liquid chromatography gradient (e.g., 5–10 min) for the characterization of protein interactions. We recently developed a long-gradient (e.g., 90 min), sub-zero temperature, ultra-high performance liquid chromatography HDX-MS (UPLC-HDX-MS) platform that has significantly increased separation power and limited back-exchange for the analysis of protein samples with high complexity. In this study, we demonstrated the utility of this platform for mapping antibody–antigen epitopes by examining four fully human monoclonal antibodies to anthrax PA. Antibody p1C03, with limited neutralizing activity in vivo, bound to a region on domain 1A of PA. p6C04 and p1A06, with no neutralizing activities, bound to the same helix on domain 3 to prevent oligomerization of PA. We found p6C01 strongly bound to domain 3 on a different helix region. We also identified a secondary epitope for p6C01, which likely leads to the blocking of furin cleavage of PA after p6C01 binding. This novel binding of p6C01 results in highly neutralizing activity. This is the first report of this distinct binding mechanism for a highly neutralizing fully human antibody to anthrax protective antigen. Studying such epitopes can facilitate the development of novel therapeutics against anthrax.

## 1. Introduction

*Bacillus anthracis* has long been recognized as a potential bioterrorist threat due to the transmissibility of its spores and the high mortality rate from inhalational infection. Such fears were realized in 2001, when spores circulated by a bioterrorist through the U.S. postal system resulted in 22 cases of inhalational anthrax and five deaths. *Bacillus anthracis* produces toxins that suppress the host’s immune system, allowing the bacteria to quickly multiply, leading to sepsis [1]. Anthrax produces two toxins, lethal toxin and edema toxin, via a tripartite system of three proteins, protective antigen (PA), lethal factor (LF), and edema factor (EF). Both LF and EF require PA to gain entry into the cell before intoxication; thus, PA is the target of immunization and passive immunotherapies for anthrax infection [2].

Intoxication proceeds by PA first binding the cell surface via its receptors CMG-2 and TEM-8 [3]. Furin-like proteases on the cell surface then cleave the 83 kDa PA into PA-63, which remains bound to the receptor, and PA-20, which dissociates from the complex. After loss of PA-20, PA-63 oligomerizes to form a heptameric or octameric structure [4]. This oligomerized PA surface can then bind LF and/or EF to form toxin, and the entire complex is internalized by the cell. After endocytosis, enzymatic toxins LF and EF are released into the cytosol. 

PA is a well-characterized protein with four functional domains. Domain 1 contains the furin cleavage site [5]. After cleavage, domain 1 becomes domain 1A (PA-20), which leaves the cell surface, and domain 1B (or 1’), which along with domain 2 forms the surface for LF and EF to bind. Domain 3 is necessary for oligomerization, and domain 4 contains the receptor-binding domain where PA binds to TEM8/CMG2. 

Antibodies to PA can prevent intoxication by a variety of mechanisms. Domain 1A is the most immunogenic portion of the protein, but antibodies directed toward it are typically unable to neutralize toxin, with a few notable exceptions [6,7]. Antibodies to domain 3 can interrupt oligomerization, but such antibodies may or may not be neutralizing [8,9]. Antibodies of most interest for immunotherapeutics bind either to domain 4 and prevent PA from binding to the cell surface, or to domain 1b/2, which either prevents EF/LF from binding and forming the toxin complex or prevents furin cleavage [10]. 

Hydrogen–deuterium exchange mass spectrometry (HDX-MS) has long been utilized as a tool for characterizing both protein dynamics and protein–protein interactions [11]. HDX-MS measures the exchange rate of protein backbone amide hydrogens with deuterium upon the exposure of protein to deuterium oxide (D_2_O) [12]. The rate of exchange for protein amide hydrogens depends on the involvement of both hydrogen bonding and solvent accessibility. When a protein binds to other protein(s), the exchange rate of the amide hydrogen in the binding regions is significantly decreased due to the reduced solvent accessibility, resulting in less deuterium incorporation in these regions. Therefore, the identified regions with the decreased deuterium incorporation can be indicative of binding site for the protein–protein interaction. Unlike the short liquid chromatography gradient times (e.g., 5–10 min) typically used in the routine HDX-MS studies [13,14,15,16,17], we have recently developed a subzero-temperature, long gradient, ultrahigh pressure liquid chromatography system located in a low-cost refrigerator with significantly improved separation power while limiting back-exchange for HDX-MS to separate deuterated protein fragments with high complexity [18]. 

Here, we demonstrate its utility for mapping antibody–antigen epitopes by examining four full-length, fully human monoclonal antibodies to anthrax PA. The first antibody, p1C03, binds to a flexible loop region on domain 1A of PA. Two of these antibodies, p6C04 and p1A06, bind to the same epitope in domain 3 in a manner that prevents oligomerization but is not neutralizing. Finally, p6C01 is a highly neutralizing antibody that prevents furin cleavage, also binding to an epitope in domain 3. Thus, we show here that antibodies binding to domain 3 in adjacent helices can have vastly different functional anti-toxin characteristics. This information will contribute to the successful development of novel therapeutics as well as novel subunit vaccines against anthrax. 

## 2. Results

In a previous work, we isolated antibody-secreting cells from a single donor after booster vaccination with AVA and generated a suite of fully human monoclonal antibodies (hmAb) with high specificity for protective antigen (PA) [9]. However, only a few of the antibodies were able to efficiently neutralize lethal toxin, measured in both a standard in vitro lethal toxin neutralization assay and in vivo mouse model, where A/J mice were challenged with lethal toxin after administration of antibody. Here, we applied and optimized the previously developed subzero UPLC-HDX-MS platform to characterize the interaction between PA and four previously isolated monoclonal antibodies to provide new insights in the neutralization response to AVA.

**Figure 1:**
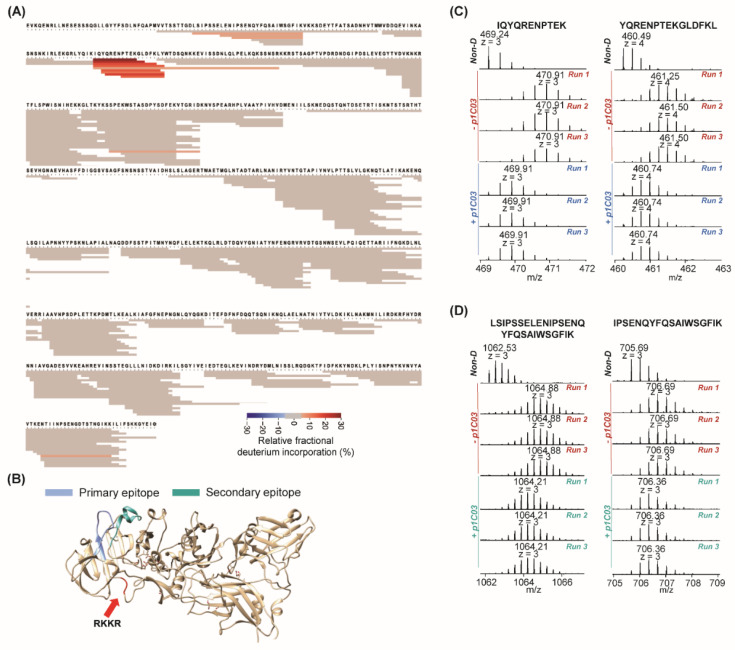
HDX-MS results for p1C03 binding. (**A**) Heatmap for epitope mapping of PA in the presence of p1C03. Peptide relative fractional deuterium incorporation (%) was calculated by subtracting the fractional deuterium incorporation of the peptides in the PA:p1C03 complex from the fractional deuterium incorporation of the peptides in free PA. The relative fractional deuterium incorporation of each fragment was indicated using the gradient in the figure legend. The peptides with the relative fractional deuterium incorporation in the range of −5% to 5% between free PA status and PA:p1C03 complex status are in grey, indicating no protection. The peptides with the relative fractional deuterium incorporation above 5% are in orange, indicating protection. (**B**) Highlights of mapped epitope in crystal structure of PA. The primary epitope is in blue. The secondary epitope is in teal. The furin cleavage site is in red. (**C**) MS spectra in triplicate of two peptides in primary epitope in the absence and presence of p1C03. (**D**) MS spectra in triplicate of two peptides in secondary epitope in the absence and presence of p1C03.

The first antibody we evaluated is p1C03. Our previous study determined that it (1) strongly binds to domain 1A and whole PA with strong binding affinity (K_d_ < 0.1 nM); (2) does not neutralize toxin in vitro; yet surprisingly (3) confers 40% protection in vivo [9]. Figure 1A shows the fragment coverage for PA after incubation of the p1C03/PA complex with D_2_O followed by protease digestion. Peptides from PA (n = 272) achieved 91% sequence coverage for epitope mapping against p1C03. Peptides colored in red showed lower relative fractional deuterium incorporation, indicating protection of those fragments from deuterium exchange in the antibody/PA complex. Two regions with relatively lower fractional deuterium uptake upon binding were highlighted on the crystal structure of PA in Figure 1B. The primary epitope showing higher protection was in light blue, and the secondary epitope with less protection was in cyan. The furin cleavage site was in red. Figure 1C shows examples of two fragments in triplicate with and without antibody in the primary epitope (IQYQRENPTEK, z = 3; YQRENPTEKGLDFKL, z = 4). Figure 1D shows two fragments from the secondary epitope (LSIPSSELENIPSENQYFQSAIWSGFIK, z = 3; IPSENQYFQSAIWSGFIK, z = 3). Spectra from all fragments in mapped epitope regions from the p1C03 experiments are presented in Supplementary Material Figure S1. The calculated average deuterium uptake of each identified PA peptide in the absence and presence of p1C03 is presented in Supplementary Table S1.

**Figure 2:**
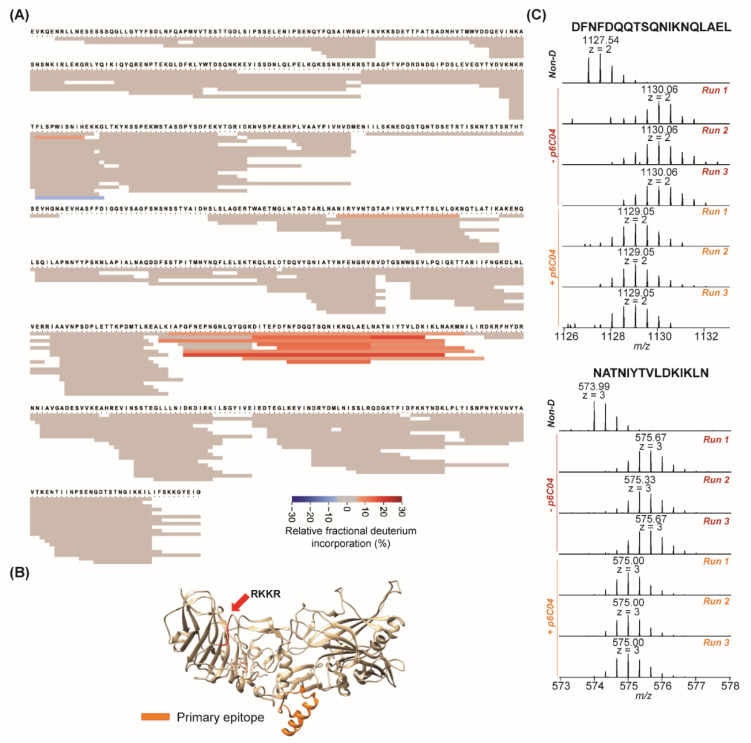
HDX-MS results for p6C04 binding. (**A**) Heatmap for epitope mapping of PA in the presence of p6C04. Peptide relative fractional deuterium incorporation (%) was calculated by subtracting the fractional deuterium incorporation of the peptides in the PA:p6C04 complex from the fractional deuterium incorporation of the peptides in free PA. The relative fractional deuterium incorporation of each fragment was indicated using the gradient in the figure legend. The peptides with the relative fractional deuterium incorporation in the range of −5% to 5% between free PA status and PA:p6C04 complex status are in grey, indicating no protection. The peptides with the relative fractional deuterium incorporation above 5% between free PA status and PA:p6C04 complex status are in orange, indicating protection. (**B**) Highlights of mapped epitope in crystal structure of PA. The primary epitope is in orange. The furin cleavage site is in red. (**C**) MS spectra in triplicate of two peptides in primary epitope in the absence and presence of p6C04.

The second antibody we evaluated is p6C04. We had previously determined that it (1) bound to recombinant domain 3 and whole PA with high affinity (K_d_ = 0.11 nM); (2) prevented oligomerization as determined by a standard SDS-PAGE oligomerization assay; yet (3) had no neutralization capacity, either in vitro in a lethal toxin neutralization assay or in vivo in a mouse lethal toxin challenge model [9]. Figure 2A shows the fragment coverage for PA after incubation of the p6C04/PA complex with D_2_O followed by protease digestion. Peptides from PA (*n* = 223) achieved 95% sequence coverage for epitope mapping against p6C04. Peptides colored in red show lower relative fractional deuterium incorporation, indicating protection of those fragments from deuterium exchange in the antibody/PA complex. These fragments with lower relative fractional deuterium incorporation are highlighted on the crystal structure of PA in Figure 2B. The highlighted area is a flexible helix on domain 3, and it would not be surprising that an antibody bound to this area could sterically hinder oligomerization. Figure 2C shows spectra for two contiguous fragments (DFNFDQQTSQNIKNQLAEL, z = 2; NATNIYTVLDKIKLN, z = 3) of PA identified as having decreased relative fractional deuterium incorporation. These spectra were obtained under conditions of no deuteration (top), with deuteration but no antibody in triplicate (middle), and with deuteration and antibody also in triplicate (bottom). In both cases, the measured decrease in deuterium incorporation was obvious and repeatable. For all of the 14 peptides identified in this epitope, a significant lower relative fractional deuterium incorporation was observed upon antibody binding (all fragments identified from the p6C04 binding epitope are presented in Supplementary Material Figure S2). The calculated average deuterium uptake of each identified PA peptide in the absence and presence of p6C04 is presented in Supplementary Table S1.

**Figure 3:**
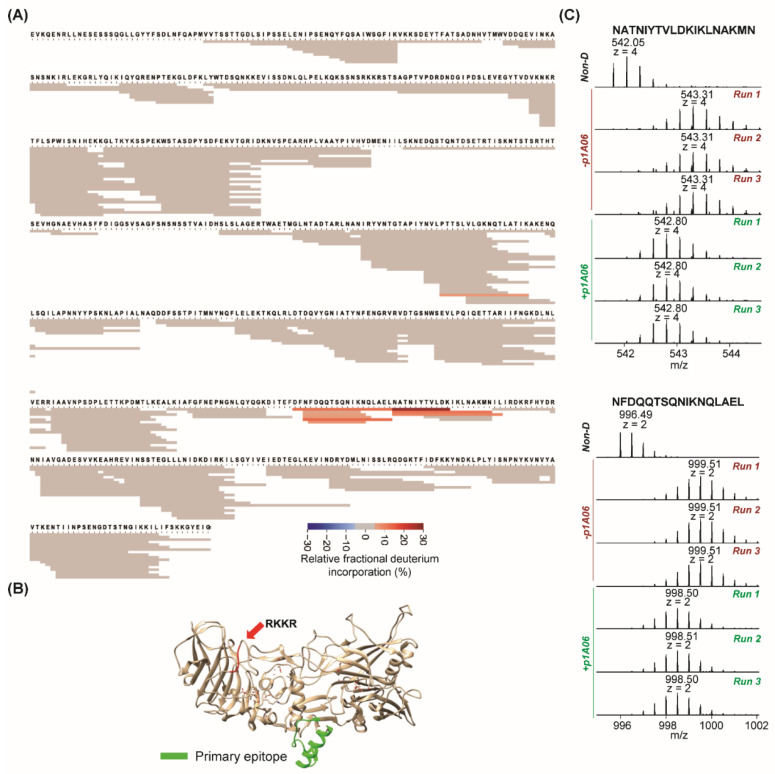
HDX-MS results for p1A06 binding. (**A**) Heatmap for epitope mapping of PA in the presence of p1A06. Peptide relative fractional deuterium incorporation (%) was calculated by subtracting the fractional deuterium incorporation of the peptides in the PA:p1A06 complex from the fractional deuterium incorporation of the peptides in free PA. The relative fractional deuterium incorporation of each fragment was indicated using the gradient in the figure legend. The peptides with the relative fractional deuterium incorporation in the range of −5% to 5% between free PA status and PA:p1A06 complex status are in grey, indicating no protection. The peptides with the relative fractional deuterium incorporation above 5% are in orange, indicating protection. (**B**) Highlights of mapped epitope in crystal structure of PA. The primary epitope is green. The furin cleavage site is in red. (**C**) MS spectra in triplicate of two peptides in primary epitope in the absence and presence of p1A06.

The third antibody we evaluated is p1A06. Our previous study found that p1A06 was similar to p6C04 with respect to binding to domain 3 and inhibiting oligomerization [9]. Figure 3A shows the fragment coverage for PA after incubation of the p1A06/PA complex with D_2_O followed by protease digestion. Peptides from PA (*n* = 269) achieved 91% sequence coverage for epitope mapping against p1A06. The region with relatively lower fractional deuterium uptake upon binding is highlighted in green on the crystal structure of PA in Figure 3B. The same epitope for p1A06 binding was identified in domain 3 as for p6C04 binding (Figure 1B and Figure 3B). Figure 3C shows spectra for two contiguous fragments (NFDQQTSQNIKNQLAEL, z = 2; NATNIYTVLDKIKLNAKMN, z = 4) of PA identified as having low relative fractional deuterium incorporation upon p1A06 binding. Spectra from all fragments in mapped epitope regions from the p1A06 experiments are presented in Supplementary Material Figure S3. The calculated average deuterium uptake of each identified PA peptide in the absence and presence of p1A06 is presented in Supplementary Table S1.

**Figure 4:**
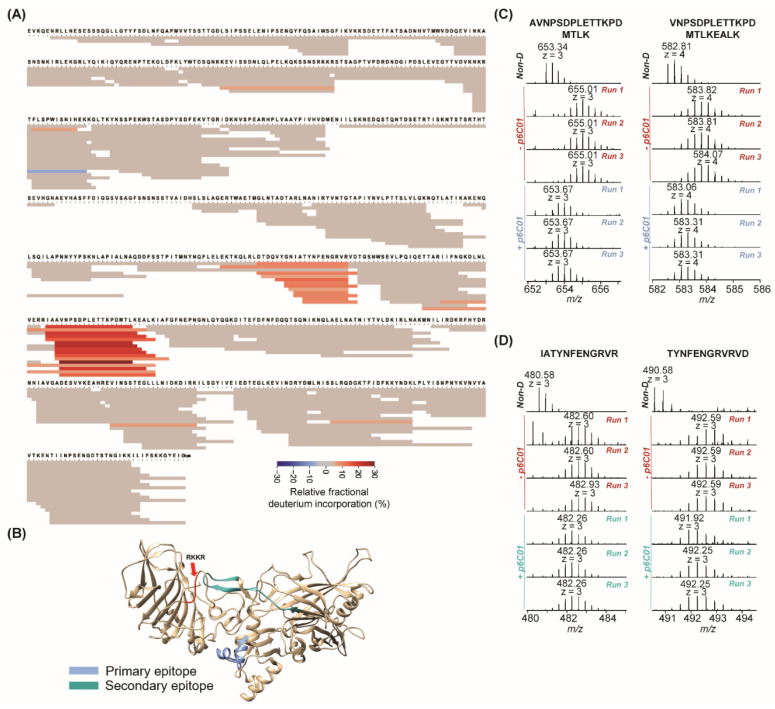
HDX-MS results for p6C01 binding. (**A**) Heatmap for epitope mapping of PA in the presence of p6C01. Peptide relative fractional deuterium incorporation (%) was calculated by subtracting the fractional deuterium incorporation of the peptides in the PA:p6C01 complex from the fractional deuterium incorporation of the peptides in free PA. The relative fractional deuterium incorporation of each fragment was indicated using the gradient in the figure legend. The peptides with the relative fractional deuterium incorporation in the range of −5% to 5% between free PA status and PA:p6C01 complex status are in grey, indicating no protection. The peptides with the relative fractional deuterium incorporation above 5% are in orange, indicating protection. (**B**) Highlights of mapped epitope in crystal structure of PA. The primary epitope is blue. The secondary epitope is teal. The furin cleavage site is in red. (**C**) MS spectra in triplicate of two peptides in primary epitope in the absence and presence of p6C01. (**D**) MS spectra in triplicate of two peptides in secondary epitope in the absence and presence of p6C01.

The last antibody we evaluated was p6C01. We were previously unable to map the binding of p6C01 other than to whole PA with high affinity (<0.1 nM) but showed that it prevented furin cleavage and was highly neutralizing both in vitro and in vivo (80% survival) [9]. We assumed that this antibody either bound a complex conformational epitope that we were unable to measure using recombinant domains, or bound to domain 1B/2, which we were unable to express. Figure 4A shows the fragment coverage for PA after incubation of the p6C01/PA complex with D_2_O followed by protease digestion. Peptides from PA (*n* = 244) achieved 95% sequence coverage for epitope mapping against p6C01. This antibody presented two surprises. First, similarly to p6C04 and p1A06, p6C01 strongly protects an exposed portion of domain 3. However, there was also a secondary epitope showing less (but highly repeatable) relative fractional deuterium incorporation in domain 2. In Figure 4B, the primary epitope is shown in blue, the secondary epitope in teal, and the furin cleavage site in red. Figure 4C shows examples of two fragments, again in triplicate with and without antibody, in the primary epitope (AVNPSDPLETTKPDMTLK, z = 3; VNPSDPLETTKPDMTLKEALK, z = 4). Figure 4D shows two fragments from the secondary epitope (IATYNFENGRVR, z = 3; TYNFENGRVRVD, z = 3). Spectra from all fragments in mapped epitope regions from the p6C01 experiments are presented in Supplementary Material Figure S4. The calculated average deuterium uptake of each identified PA peptide in the absence and presence of p6C01 is presented in Supplementary Table S1.

## 3. Discussion

Examination of the epitope binding of these four monoclonal antibodies both highlights the importance of understanding the relationship between epitope binding and functional characteristics of the antibodies in question and emphasizes how HDX technology can be used to accomplish this goal. While the data we obtained for these monoclonal antibodies overlay the various functional and binding assays previously found to characterize them, HDX-MS revealed more precise targeting involved in PA’s secondary structure for these antibodies from AVA-induced immune responses. Taken together, these mapped epitopes associated with the neutralization activities and functions (Table 1) will facilitate the understanding of the elicited human humoral immune response after AVA vaccination and will contribute to the successful development of antibody cocktail-based novel therapeutics as well as novel vaccines against anthrax.

Antibody p6C01 presented several surprises. Our HDX results show that this antibody clearly binds to domain 3, but with a secondary protected region with lower relative fractional deuterium incorporation in domain 2. While we are confident of its presence, further research is necessary to determine the exact nature of this region. It could form a legitimate interaction with a section of the antibody away from the CDRs participating in the primary binding, or perhaps simply strong binding to the primary epitope positions the antibody in a way that also weakly protects the protected region by steric hindrance. It is also possible that the binding of the antibody causes a conformational change in PA, moving the primary and secondary epitopes into close proximity of each other. Keeping in mind that this antibody neutralizes by preventing furin cleavage, it seems likely that the portion of the antibody protecting the secondary region is also blocking the furin cleavage site, whether sterically or by conformational change. There are previous reports of monoclonal antibodies that blocked furin cleavage while binding away from the furin cleavage site, even from domain 1A [6]. However, this is the first documented example demonstrating this manner of binding of a highly neutralizing fully human antibody to anthrax-protective antigen domain 3 in a manner that prevents furin cleavage. 

Our work also suggests that experiments with recombinant protein domains should be used with understanding of their limitations. Either the GST tag interfered with p6C01 binding to the recombinant domain 3 fragment through steric hindrance or preventing proper folding of the domain, or binding of the secondary epitope is necessary for its extremely high affinity interaction with full-length PA. The ability of HDX-MS to measure complex conformational epitopes from native, properly folded protein is a strength of this technique.

We also show that p6C04 and p1A06 bind to the same epitope of domain 3. It is important to point out that these two antibodies are not clonally related, and in fact, p1A06 uses VH5-51*03/JH6*02 and has a remarkably long 26 a.a. CDR3, while p6C04 uses VH4-59*03/JH5*02 with a 20 a.a. CDR3. Although both utilizing VK1 (p1A06: VK1-16*02; p6C04: VK1-5*04), the light chains also have quite dissimilar CDR3s. We point out these differences for several reasons: (1) it is clear that certain portions of PA are more immunogenic eliciting diverse antibody responses to the same epitope, (2) the total number of epitopes on PA that different antibodies might bind could be quite limited. We are further investigating this phenomenon.

## 4. Conclusions

In summary, we have reported our adaptations of HDX-MS to be able to accomplish long gradient UPLC separations with limited back-exchange for handling protein samples with high complexity [18]. Here, we applied this technology to four fully human monoclonal antibodies and mapped their binding to anthrax protective antigen. Similar to p6C04 and p1A06, p6C01 strongly binds to domain 3, but it presents entirely different neutralizing characteristics. The characterized secondary epitope for p6C01 binding suggested the allosteric effect on monoclonal antibody neutralization, which highlights the utilization of this technology for providing valuable conformational information on antibody/antigen binding without the need for expensive or time-consuming crystallography or cryo-EM techniques.

## 5. Materials and Methods

### 5.1. Materials and Reagents

All chemicals, including Protease Type XIII from *Aspergillus saitoi* (≥0.6 unit/mg) and deuterium oxide (≥99.6 atom % D), were purchased from Sigma-Aldrich (Milwaukee, WI, USA) unless noted otherwise. An ACE^®^ Excel^®^ SuperC18™ column (100 mm × 2.1 mm, 1.7 μm, 90 Å) was purchased from Advanced Chromatography Technologies Ltd. (Aberdeen, Scotland). Recombinant PA was purchased from List Biological Laboratories, Inc. (Campbell, CA, USA). Fully human monoclonal antibodies p6C01, p1C03, p1A06, and p6C04 were produced in HEK293 cells as previously described [9,19]. 

### 5.2. Differential UPLC-HDX-MS

Differential HDX-MS experiments were performed for PA epitope mapping. For epitope mapping on antibodies p6C04 and p6C01, protein samples were prepared as 6 μM PA alone, 6 μM PA premixed with p6C01 antibody at molar ratio of 1:1, or 6 μM PA premixed with p6C04 antibody at molar ratio of 1:1. Free PA and PA premixed with p6CO4 were first used to optimize HDX reaction conditions. A total of 4 μL of free PA sample and 4 μL of PA:p6C04 complex sample were diluted with 36 μL of 1× PBS in D_2_O and incubated for various times (2 min, 5 min, and 10 min) at room temperature. In this HDX reaction, 2 min deuterium labeling exhibited the distinguishable relative fractional deuterium incorporation between PA alone and PA bound with p6C04 (Figure S5). In order to increase throughput for long gradient UPLC-HDX-MS analysis, we used a single shorter incubate time of 2 min for other three antibodies’ epitope mapping. A total of 4 μL of PA:p6C01 complex sample was then diluted with 36 μL of 1× PBS in D_2_O and incubated for 2 min at room temperature. Experiments for each sample were conducted in triplicate. The reaction was quenched by adding 40 μL of chilled 50% acetonitrile in 1% formic acid to a final pH of 2.5 and incubated at 0 °C for 2 min. The quenched solution then was incubated with 80 μL of 2.4 mg/mL protease type XIII at 0 °C for 4 min for protein digestion. The digested peptides were quickly injected into the subzero temperature UPLC system (0.1% formic acid and 10% acetonitrile in water for mobile phase A, and 0.1% formic acid in acetonitrile for mobile phase B) with a gradient from 0% to 33% mobile phase B at 150 μL/min flowrate over 30 min at −10 °C, followed by mass spectrometer detection [18]. A total of 4 μL of non-deuterated 6 μM PA sample was incubated with 36 μL of 1× PBS in H_2_O, followed by same quenching, digestion, subzero temperature UPLC separation, and MS analysis steps for peptide identification. 

For epitope mapping on antibodies p1A06 and p1C03, protein samples were prepared as 2.7 μM PA alone, 2.7 μM PA premixed with p1A06 antibody at molar ratio of 1:1, or 2.7 μM PA premixed with p1C03 antibody at molar ratio of 1:1. For HDX reactions, 9 μL of free PA sample, 9 μL of PA: p1A06 complex sample, and 9 μL of PA: p1C03 complex sample were diluted with 72 μL of 1× PBS in D_2_O and incubated for 2 min at room temperature, individually. Experiments for each sample were conducted in triplicate. The reaction was quenched by adding 80 μL of chilled 30% acetonitrile in 1% formic acid to a final pH of 2.5 and incubated at 0 °C for 2 min. The quenched solution then was incubated with 80 μL of 2.4 mg/mL protease type XIII at 0 °C for 4 min for protein digestion. The digested peptides were quickly injected into the subzero temperature UPLC system (0.1% formic acid and 10% acetonitrile in water for mobile phase A and 0.1% formic acid in acetonitrile for mobile phase B) with a gradient from 0% to 33% mobile phase B at 150 μL/min flowrate over 30 min at −10 °C, followed by mass spectrometer detection [18]. A total of 9 μL of non-deuterated 2.7 μM PA sample was incubated with 72 μL of 1× PBS in H_2_O, followed by same quenching, digestion, subzero temperature UPLC separation, and MS analysis steps for peptide identification. 

An Orbitrap Exploris 240 mass spectrometer (Thermo Fisher Scientific, Bremen, Germany) was used for LC–MS/MS. The heated capillary temperature was set to 320 °C with a spray voltage of 3.5 kV. Sheath gas and Aux gas were set to 35 L/min and 7 L/min for improving ionization, respectively. MS scans were obtained with a resolution setting of 120,000 and *m*/*z* range from 350 to 1350. The AGC for MS scans was set to standard mode, and the max ion time was set to 1000 ms with 2 micro scans. MS/MS scans were acquired with a resolution setting of 30,000 and *m*/*z* range from 150 to 2000 with higher-energy C-trap dissociation (HCD) at a normalized collision energy setting of 30%. The 10 most abundant precursor ions were selected for MS/MS. The AGC for MS/MS was set to standard mode, and the max ion injection time was set to auto mode with 2 micro scans. 

### 5.3. HDX-MS Data Analysis

The LC–MS/MS data of non-deuterated PA were searched against a database containing PA sequence using MSGF+ [20]. The decoy database is automatically generated in MSGF+ software. The peptide identifications were filtered using a SpecE value cut-off of 1 × 10^10^ (i.e., the calculated FDR <1% at the unique peptide level). The identified peptides listed together with LC–MS/MS data from all deuterated samples were then imported into the in-house-developed software to calculate the deuterium uptake levels of the identified peptides in all deuterated samples. This software fits peptide mass distributions to a Gaussian model and calculates the R^2^ value [18]. All peptides were manually checked to ensure that the correct isotopic patterns were taken into account to calculate the deuterium uptake levels of the corresponding peptides. Fractional deuterium incorporation of the identified peptides [21,22] between PA alone and the PA/antibody complex were calculated. Peptide relative fractional deuterium incorporation (%) was calculated by subtracting the fractional deuterium incorporation of the peptides in the PA/antibody complex from the fractional deuterium incorporation of the peptides in free PA [23]. Any peptide that showed a reduction in fraction deuterium incorporation of 5% or greater in the complex sample was considered to be protected [24,25]. 

## Figures and Tables

**Table 1:** Summary of four fully human monoclonal antibodies and their binding to anthrax protective antigen. In vivo neutralization (% survival) and domain activity were reported on the basis of previous studies (columns marked with *) [9]. The specific epitopes were mapped using HDX-MS in this study.

Antibody	In Vivo Neutralization (% A/JMouse Survival after Toxin Challenge) *	Domain Reactivity (ELISA) *	Functional *	Epitope(s)
p6C04	0	3	Oligomerization	DITEFDFNFDQQTSQNIKNQLAELNAT-NIYTVLDKIKLNAKMN
p1C03	40	1A	--	LSIPSSELENIPSEN; IQYQRENPTEKGLDFKLLSIPSSELENIPSEN
p1A06	20	3	Oligomerization	DITEFDFNFDQQTSQNIKNQLAELNAT-NIYTVLDKIKLNAKMN
p6C01	80	Whole PA	Furin cleavage	AVNPSDPLETTKPDMTL (in domain 3)TDQVYGNIATYN (in domain 2)

* Data from [9].

## Data Availability

The data presented in this study are available in supplementary materials.

## Supplementary Materials

The following are available online at https://www.mdpi.com/article/10.3390/toxins14020092/s1, Figure S1: MS spectra of 14 identified peptides in the primary epitope for p1C03 binding. Figure S2: MS spectra of 12 identified peptides in mapped epitopes for p6C04 binding. Figure S3: MS spectra of 9 identified peptides for p1A06 binding. Figure S4: MS spectra of 28 identified peptides in the mapped epitopes for p6C01 binding. Figure S5: HDX time-course study for PA upon p6C04 binding. Table S1: Calculated average deuterium uptake of each identified PA peptide in the absence and presence of each antibody.
