# Supplementary material for: Hydrogen-Deuterium Exchange Mass Spectrometry Reveals a Novel Binding Region of a Neutralizing Fully Human Monoclonal Antibody to Anthrax Protective Antigen
Source: Toxins (Basel). 2022 Jan 25;14(2):92. doi: 10.3390/toxins14020092 (PMC8877668; doi:10.3390/toxins14020092)
Supplement: Supplementary file 1 [file toxins-14-00092-s001.zip › Supplementary_Figures.pdf]

Supplementary Materials: Hydrogen–Deuterium Exchange Mass Spectrometry Reveals a Novel Binding Region of a Neutralizing Fully Human Monoclonal Antibody to Anthrax Protective Antigen

Mulin Fang, Zhe Wang, Kathleen Norris, Judith A. James, Si Wu and Kenneth Smith*

(A)

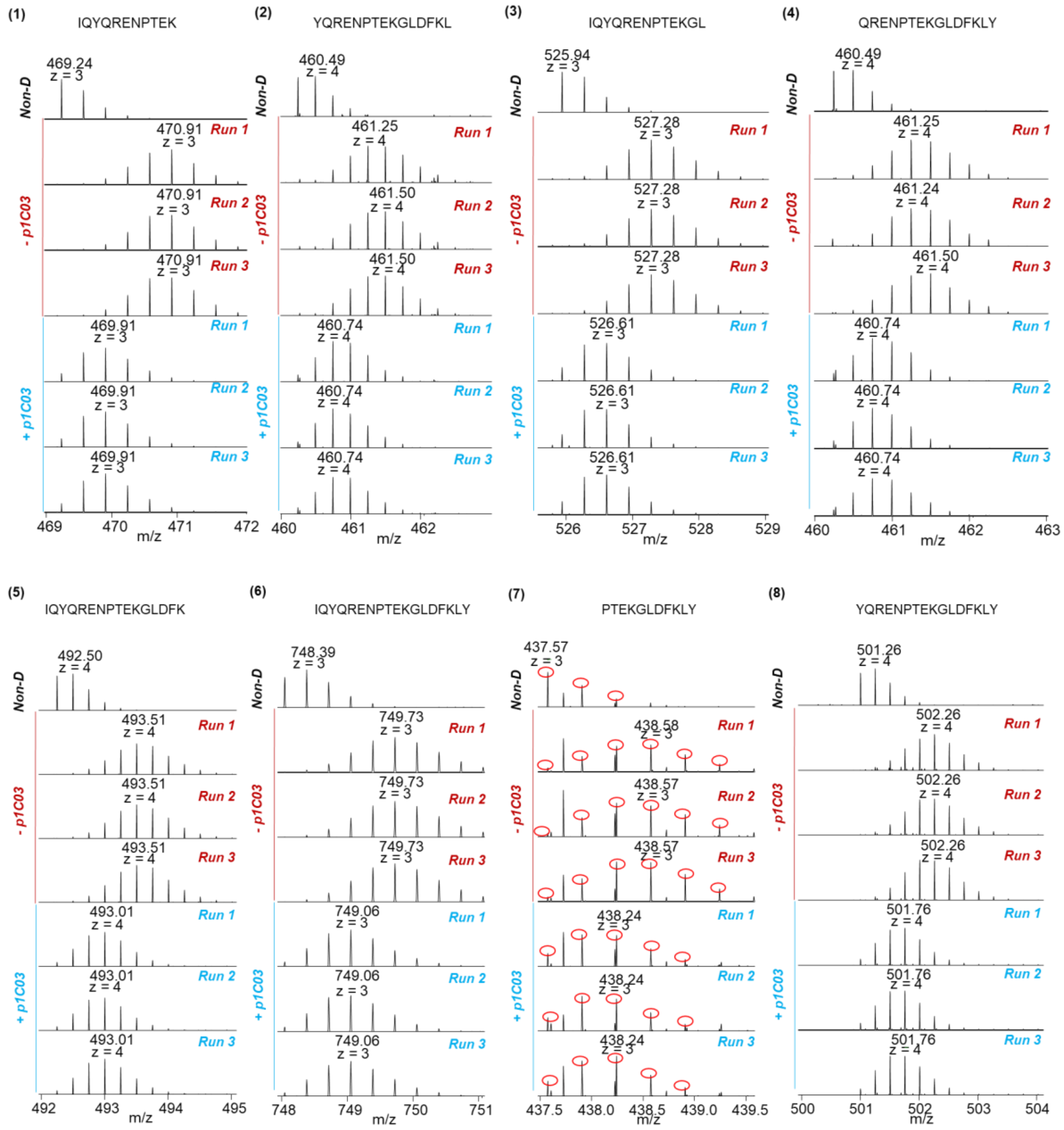

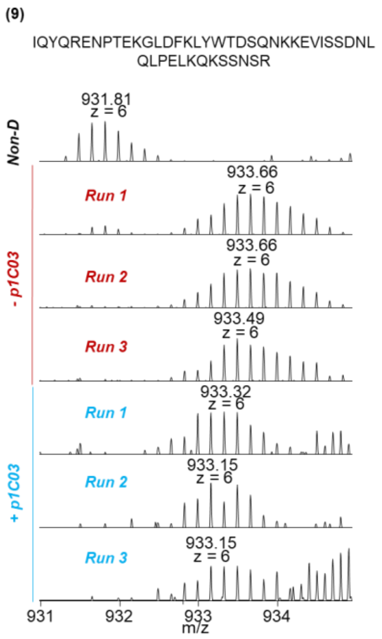

(**B**)

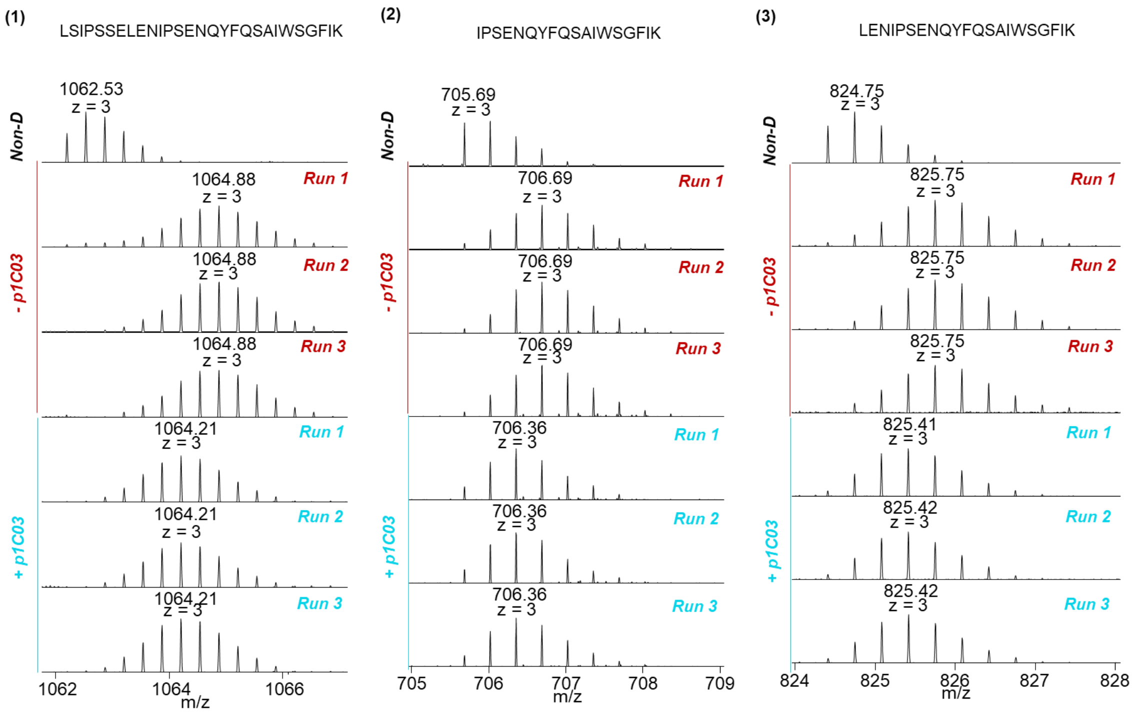

Figure S1. MS spectra of 12 identified peptides for p1C03 binding.(**A**) MS spectra of 9 identified peptides in primary epitope. (**B**) MS spectra of 3 identified peptides in secondary epitope. Red ovals highlight the correct peak distribution for peptide 7.

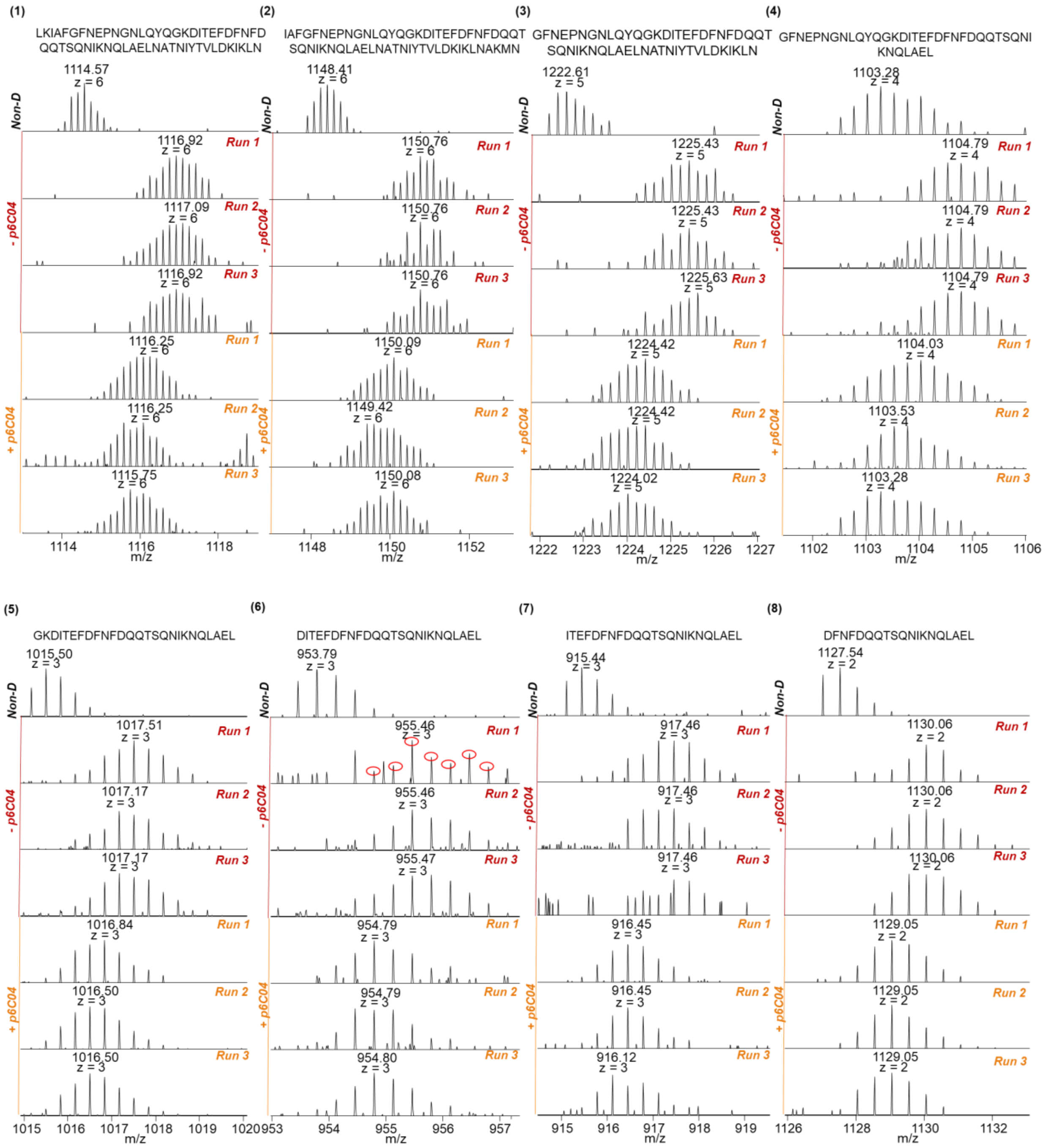

(1)
LKIAFGFNEPNGNLQYQGKDITEFDFNFD QQTSQNIKNQLAELNATNIYTVLDKIKLN
(2)
IAFGFNEPNGNLQYQGKDITEFDFNFDQQT SQNIKNQLAELNATNIYTVLDKIKLNAKMN
(3)
GFNEPNGNLQYQGKDITEFDFNFDQQT SQNIKNQLAELNATNIYTVLDKIKLN
(4)
GFNEPNGNLQYQGKDITEFDFNFDQQTSQNI KNQLAEL
(5)
GKDITEFDFNFDQQTSQNIKNQLAEL
(6)
DITEFDFNFDQQTSQNIKNQLAEL
(7)
ITEFDFNFDQQTSQNIKNQLAEL
(8)
DFNFDQQTSQNIKNQLAEL
Non-D
- p6C04
+ p6C04
Run 1
Run 2
Run 3
m/z

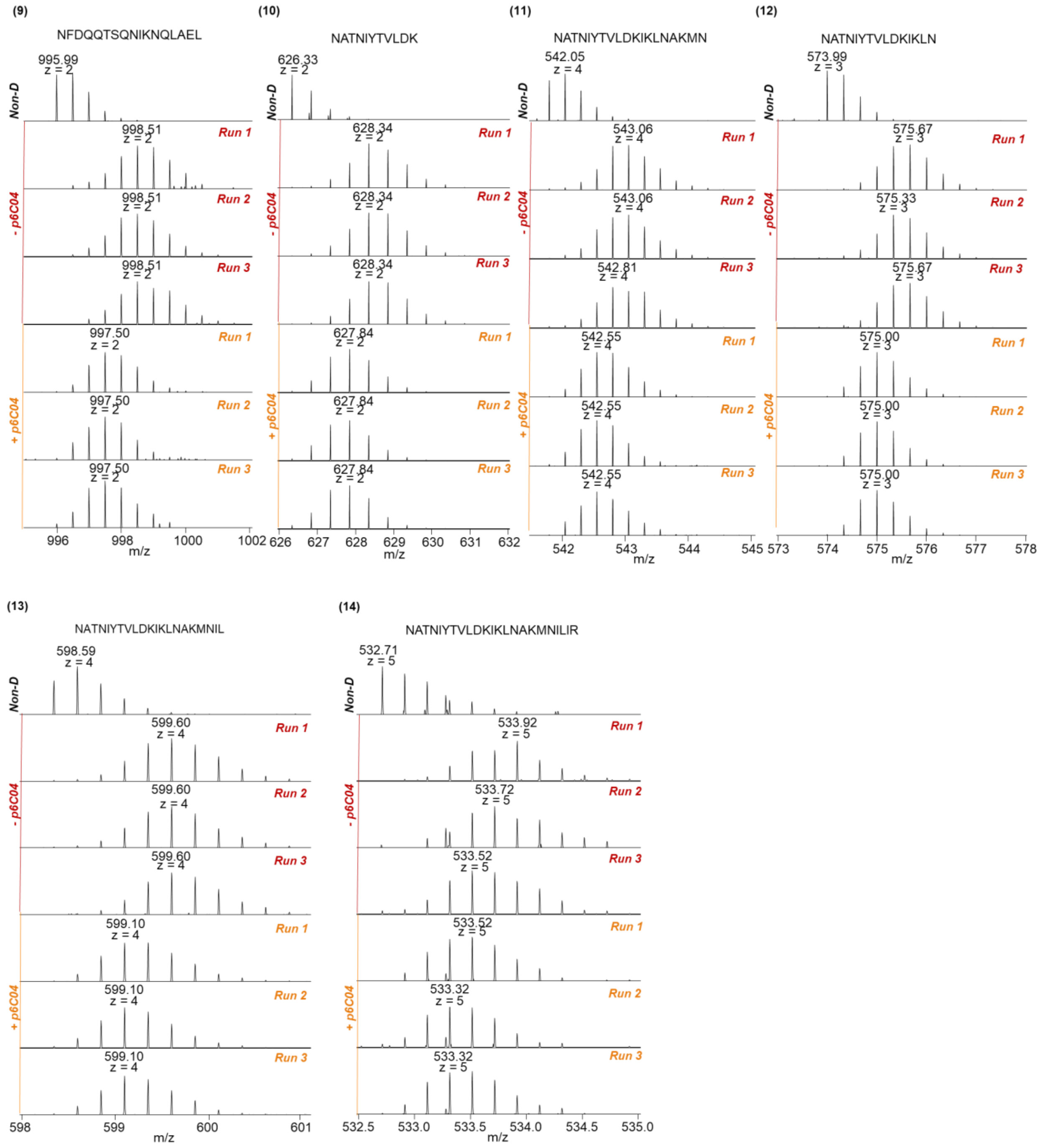

Figure S2. MS spectra of 14 identified peptides in primary epitope for p6C04 binding.

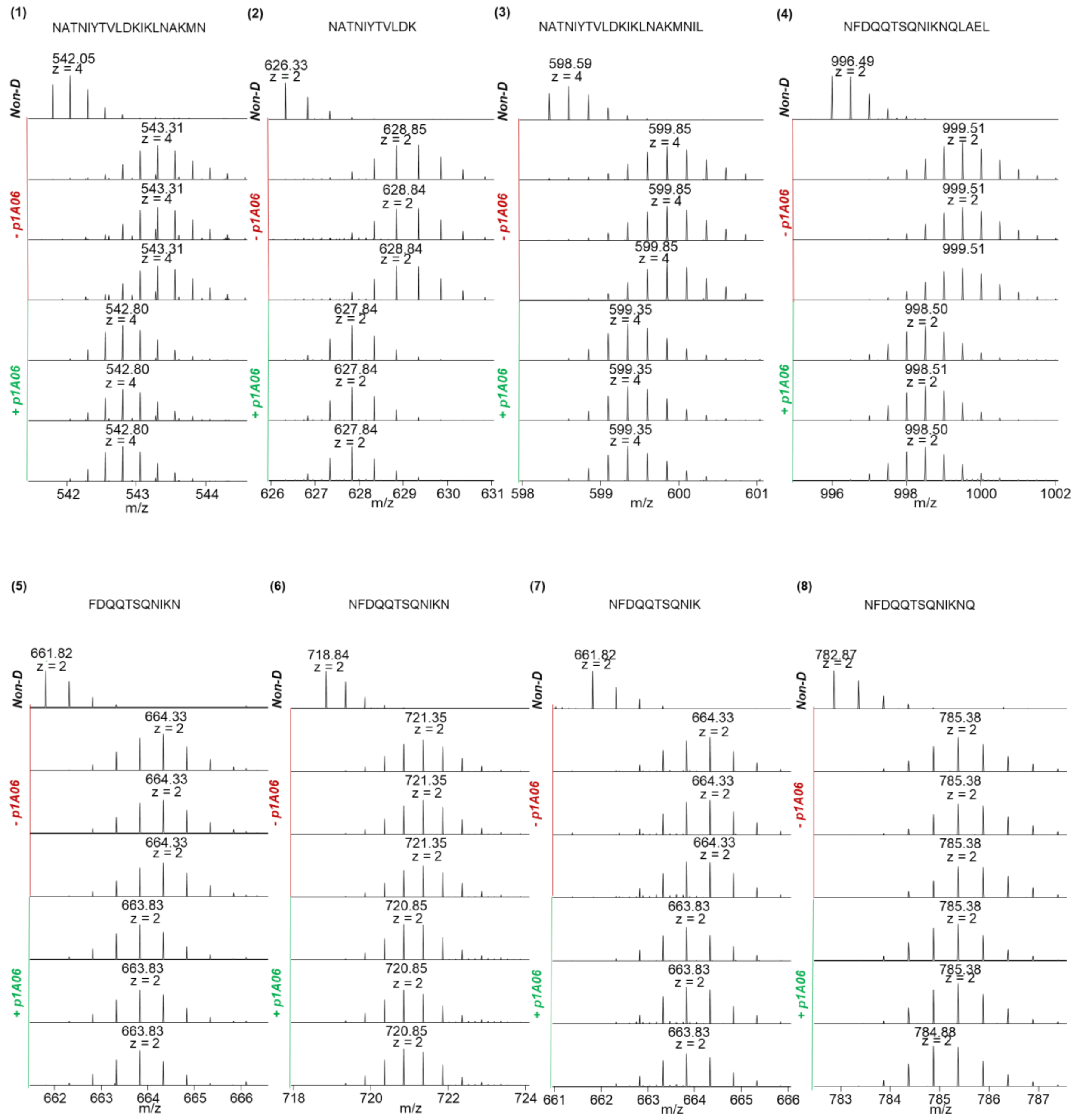
(1)
NATNIYTVLDKIKLNAKMN
(2)
NATNIYTVLDK
(3)
NATNIYTVLDKIKLNAKMNIL
(4)
NFDQQTSQNIKNQLAEL
Non-D
- p1A06
+ p1A06
m/z
(5)
FDQQTSQNIKN
(6)
NFDQQTSQNIKN
(7)
NFDQQTSQNIK
(8)
NFDQQTSQNIKNQ

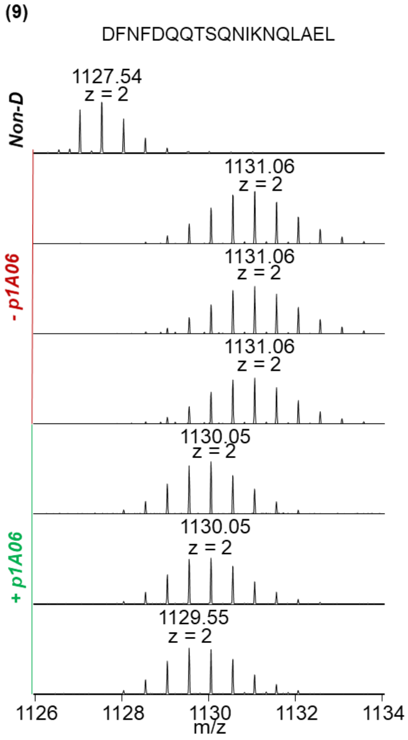

Figure S3. MS spectra of 9 identified peptides in primary epitope for p1A06 binding.

(A)

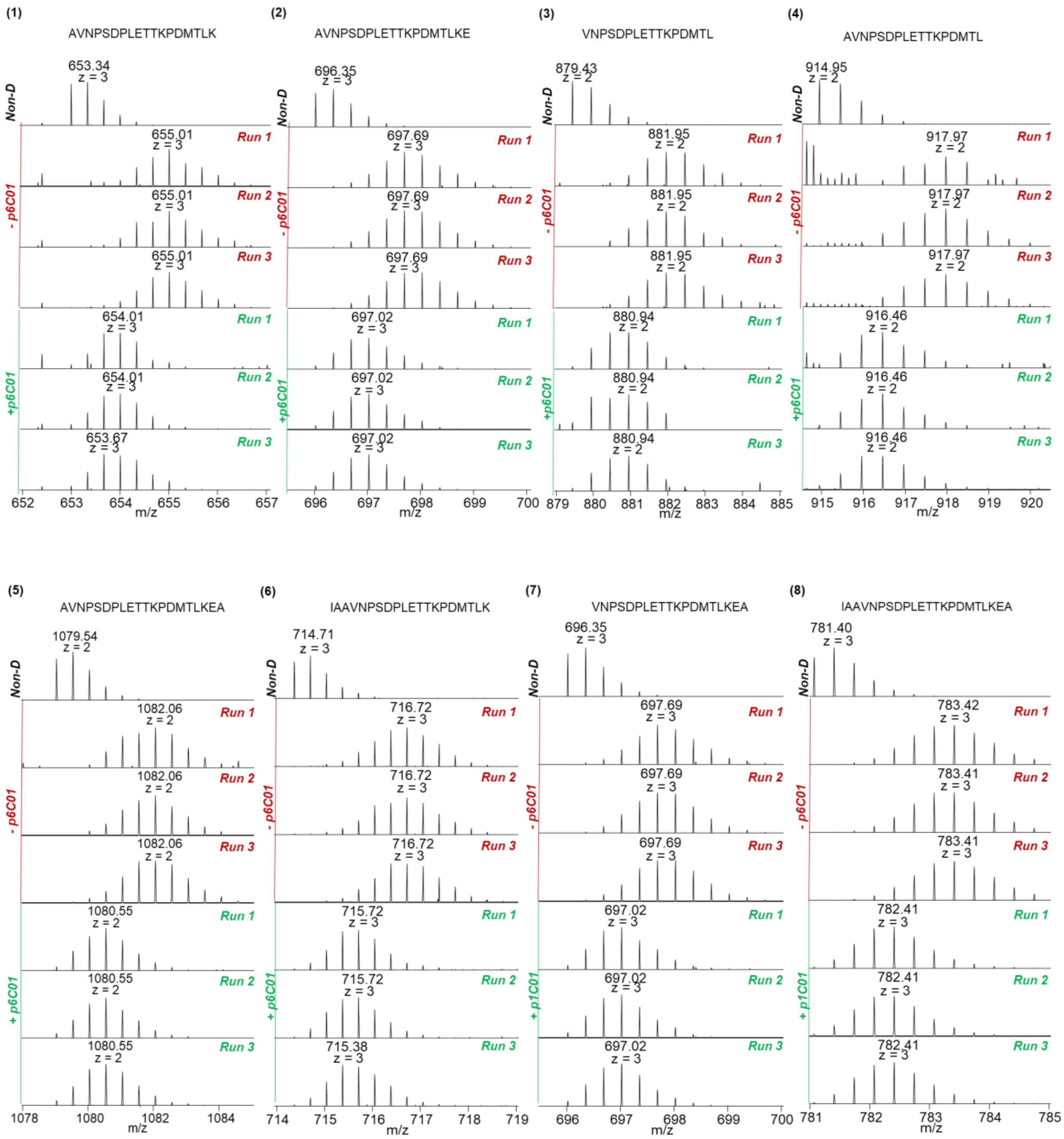
(1)
AVNPSDPLETTKPDMTLK
653.34
z = 3
655.01
z = 3
655.01
z = 3
655.01
z = 3
654.01
z = 3
654.01
z = 3
653.67
z = 3
652 653 654 655 656 657
m/z
(2)
AVNPSDPLETTKPDMTLKE
696.35
z = 3
697.69
z = 3
697.69
z = 3
697.69
z = 3
697.02
z = 3
697.02
z = 3
697.02
z = 3
696 697 698 699 700
m/z
(3)
VNPSDPLETTKPDMTL
879.43
z = 2
881.95
z = 2
881.95
z = 2
881.95
z = 2
880.94
z = 2
880.94
z = 2
880.94
z = 2
879 880 881 882 883 884 885
m/z
(4)
AVNPSDPLETTKPDMTL
914.95
z = 2
917.97
z = 2
917.97
z = 2
917.97
z = 2
916.46
z = 2
916.46
z = 2
916.46
z = 2
915 916 917 918 919 920
m/z
Non-D
- p6C01
+p6C01
Run 1
Run 2
Run 3
(5)
AVNPSDPLETTKPDMTLKEA
1079.54
z = 2
1082.06
z = 2
1082.06
z = 2
1082.06
z = 2
1080.55
z = 2
1080.55
z = 2
1080.55
z = 2
1078 1080 1082 1084
m/z
(6)
IAAVNPSDPLETTKPDMTLK
714.71
z = 3
716.72
z = 3
716.72
z = 3
716.72
z = 3
715.72
z = 3
715.72
z = 3
715.38
z = 3
714 715 716 717 718 719
m/z
(7)
VNPSDPLETTKPDMTLKEA
696.35
z = 3
697.69
z = 3
697.69
z = 3
697.69
z = 3
697.02
z = 3
697.02
z = 3
697.02
z = 3
696 697 698 699 700
m/z
(8)
IAAVNPSDPLETTKPDMTLKEA
781.40
z = 3
783.42
z = 3
783.41
z = 3
783.41
z = 3
782.41
z = 3
782.41
z = 3
782.41
z = 3
781 782 783 784 785
m/z
Non-D
- p6C01
+ p6C01
+ p1C01
Run 1
Run 2
Run 3

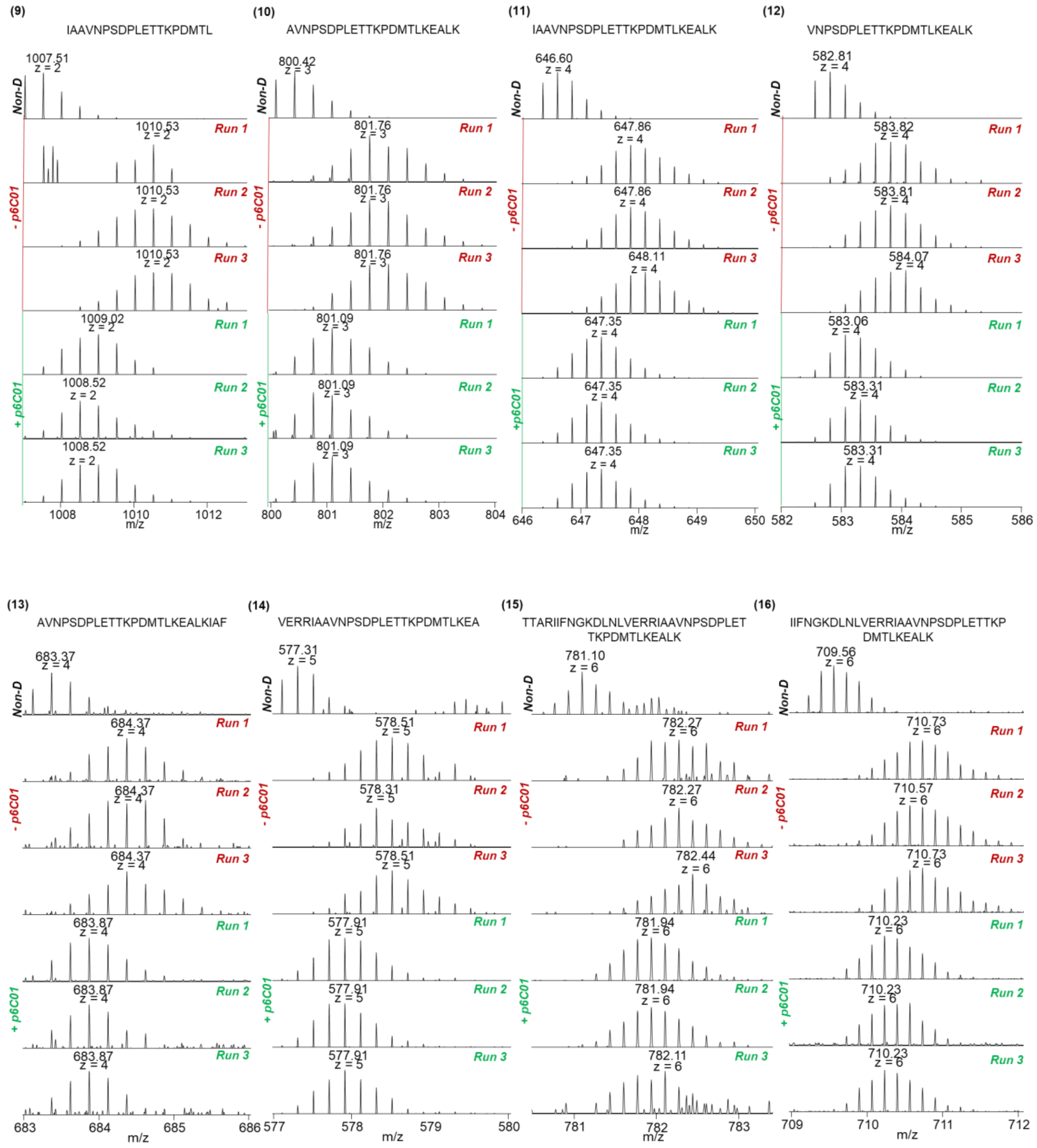

(9)
IAAVNPSDPLETTKPDMTL
1007.51 z = 2
1010.53 z = 2
1010.53 z = 2
1010.53 z = 2
1009.02 z = 2
1008.52 z = 2
1008.52 z = 2
(10)
AVNPSDPLETTKPDMTLKEALK
800.42 z = 3
801.76 z = 3
801.76 z = 3
801.76 z = 3
801.09 z = 3
801.09 z = 3
801.09 z = 3
(11)
IAAVNPSDPLETTKPDMTLKEALK
646.60 z = 4
647.86 z = 4
647.86 z = 4
648.11 z = 4
647.35 z = 4
647.35 z = 4
647.35 z = 4
(12)
VNPSDPLETTKPDMTLKEALK
582.81 z = 4
583.82 z = 4
583.81 z = 4
584.07 z = 4
583.06 z = 4
583.31 z = 4
583.31 z = 4
(13)
AVNPSDPLETTKPDMTLKEALKIAF
683.37 z = 4
684.37 z = 4
684.37 z = 4
684.37 z = 4
683.87 z = 4
683.87 z = 4
683.87 z = 4
(14)
VERRIAAVNPSDPLETTKPDMTLKEA
577.31 z = 5
578.51 z = 5
578.31 z = 5
578.51 z = 5
577.91 z = 5
577.91 z = 5
577.91 z = 5
(15)
TTARIIFNGKDLNLVERRIAAVNPSDPLET TKPDMTLKEALK
781.10 z = 6
782.27 z = 6
782.27 z = 6
782.44 z = 6
781.94 z = 6
781.94 z = 6
782.11 z = 6
(16)
IIFNGKDLNLVERRIAAVNPSDPLETTKP DMTLKEALK
709.56 z = 6
710.73 z = 6
710.57 z = 6
710.73 z = 6
710.23 z = 6
710.23 z = 6
710.23 z = 6
Non-D
- p6C01
+ p6C01
Run 1
Run 2
Run 3
m/z

(**B**)

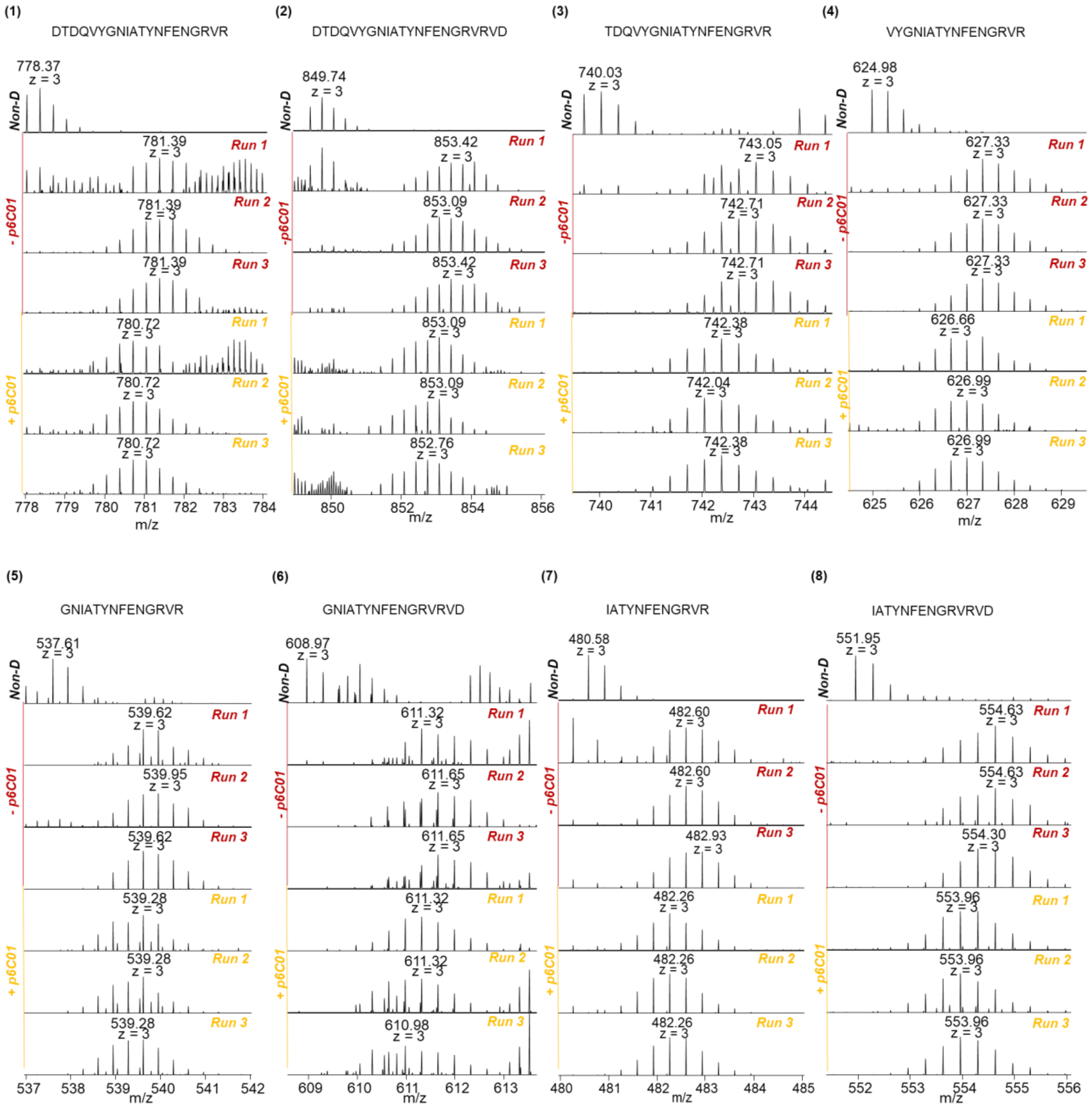
(1)
DTDQVYGNIATYNFENGRVR
778.37
z = 3
781.39
z = 3
781.39
z = 3
781.39
z = 3
780.72
z = 3
780.72
z = 3
780.72
z = 3
778 779 780 781 782 783 784
m/z
(2)
DTDQVYGNIATYNFENGRVRVD
849.74
z = 3
853.42
z = 3
853.09
z = 3
853.42
z = 3
853.09
z = 3
853.09
z = 3
852.76
z = 3
850 852 854 856
m/z
(3)
TDQVYGNIATYNFENGRVR
740.03
z = 3
743.05
z = 3
742.71
z = 3
742.71
z = 3
742.38
z = 3
742.04
z = 3
742.38
z = 3
740 741 742 743 744
m/z
(4)
VYGNIATYNFENGRVR
624.98
z = 3
627.33
z = 3
627.33
z = 3
627.33
z = 3
626.66
z = 3
626.99
z = 3
626.99
z = 3
625 626 627 628 629
m/z
(5)
GNIATYNFENGRVR
537.61
z = 3
539.62
z = 3
539.95
z = 3
539.62
z = 3
539.28
z = 3
539.28
z = 3
539.28
z = 3
537 538 539 540 541 542
m/z
(6)
GNIATYNFENGRVRVD
608.97
z = 3
611.32
z = 3
611.65
z = 3
611.65
z = 3
611.32
z = 3
611.32
z = 3
610.98
z = 3
609 610 611 612 613
m/z
(7)
IATYNFENGRVR
480.58
z = 3
482.60
z = 3
482.60
z = 3
482.93
z = 3
482.26
z = 3
482.26
z = 3
482.26
z = 3
480 481 482 483 484 485
m/z
(8)
IATYNFENGRVRVD
551.95
z = 3
554.63
z = 3
554.63
z = 3
554.30
z = 3
553.96
z = 3
553.96
z = 3
553.96
z = 3
552 553 554 555 556
m/z
Non-D
- p6C01
+ p6C01
Run 1
Run 2
Run 3

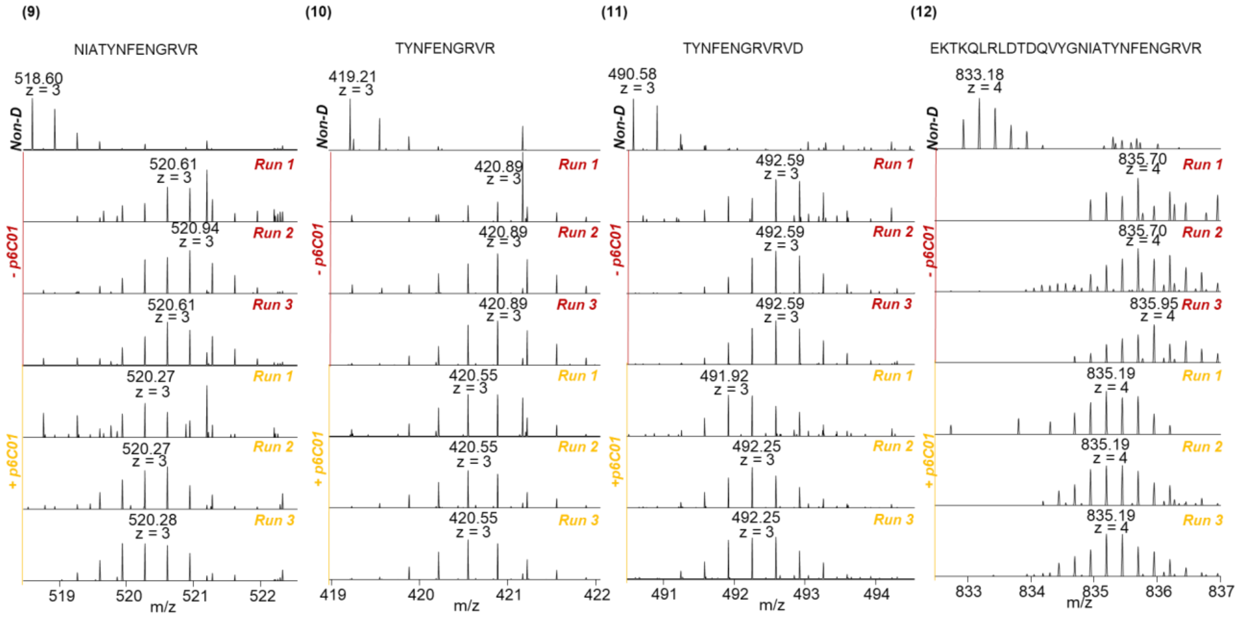

Figure S4. MS spectra of 28 identified peptides for p6C01 binding.(**A**) MS spectra of 16 identified peptides in primary epitope. (**B**) MS spectra of 12 identified peptides in secondary epitope.

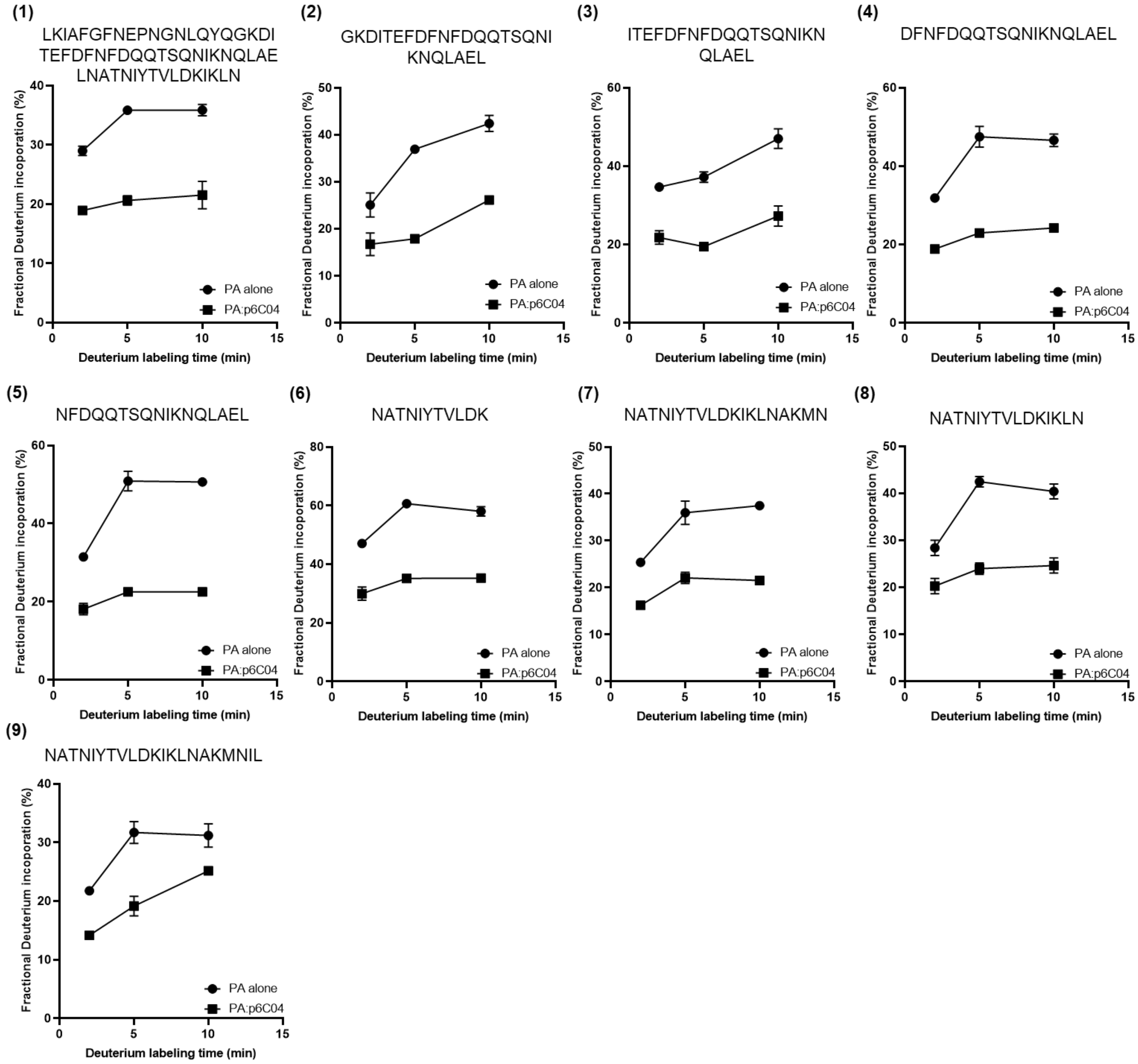

Figure S5. HDX time-course study for PA upon p6C04 binding. Relative fractional deuterium incorporation for 9 peptides in binding region were compared in the absence (Free PA) and presence of p6C04 (PA:p6C04) under 2 minutes, 5 minutes, and 10 minutes deuterium labeling conditions. Experiments were performed in triplicate.
